# Genetic variation in N-use efficiency and associated traits in Indian wheat cultivars

**DOI:** 10.1016/j.fcr.2018.06.002

**Published:** 2018-08-01

**Authors:** A.S. Nehe, S. Misra, E.H. Murchie, K. Chinnathambi, M.J. Foulkes

**Affiliations:** a Division of Plant and Crop Science, School of Biosciences University of Nottingham, Sutton Bonington, Loughborough, Leicestershire LE12 5RD, UK; b Genetics and Plant Breeding Department, Agharkar Research Institute, Pune 411 004, Maharashtra, India

**Keywords:** Nitrogen-use efficiency, N uptake, Leaf senescence, Wheat, Breeding

## Abstract

Nitrogen (N) fertilizer represents a significant cost for the grower and may also have environmental impacts through nitrate leaching and N_2_O (a greenhouse gas) emissions associated with denitrification. The objectives of this study were to quantify the genetic variability in N-use efficiency (NUE) in Indian spring wheat cultivars and identify traits for improved NUE for application in breeding. Twenty eight bread wheat cultivars and two durum wheat cultivars were tested in field experiments in two years in Maharashtra, India. Detailed growth analysis was conducted at anthesis and harvest including dry matter (DM) and N partitioning. Senescence of the flag leaf was assessed from a visual score every 3–4 days from anthesis to complete flag-leaf senescence and fitted against thermal time to estimate the onset and end of post-anthesis senescence. Grain yield (GY) was reduced under low N (LN) by an average of 1.46 t ha^−1^ (−28%). Significant N × genotype level interaction was observed for grain yield and NUE. Above-ground N uptake at harvest was reduced from 162 kg N ha^−1^ under high N (HN) to 85 kg N ha^−1^ under low N (LN) conditions, while N-utilization efficiency (grain DM yield per unit crop N uptake at harvest; NUtE) increased from 32.7 to 44.6 kg DM kg^−1^ N. Genetic variation in GY under LN related mainly to variation in N uptake at harvest rather than NUtE; and the N × genotype effect for GY was mainly explained by the interaction for N uptake at harvest. Averaging across years, the linear regression of onset of flag-leaf senescence on GY amongst cultivars was significant under both HN (R^2^ 0.16. *p *< 0.05) and LN (R^2^ 0.21, *p *< 0.05) conditions. Onset of flag-leaf senescence was positively associated with N uptake at anthesis under HN (R^2^ 0.34, p < 0.001) and LN (R^2^ 0.22, p < 0.01) conditions. Flag-leaf senescence timing was not associated with post-anthesis N uptake. It is concluded that increased N accumulation at anthesis was correlated with flag-leaf senescence timing and that N accumulation at anthesis is an important trait for enhancing grain yield and NUE of wheat grown under low to moderate N supply in India.

## Highlights

• Cultivar grain yield responses to N limitation associated with N uptake at anthesis.
• N-use efficiency in thirty wheat cultivars was correlated with timing of flag-leaf senescence.
• Flag-leaf senescence timing was correlated with N accumulation at anthesis.
• The yield in N stressed crops was limited by grain source capacity.

## 1 Introduction

Wheat (*Triticum aestivum* L.) is the most widely grown cereal crop in the world, and accounts for 20% of the calories consumed by the global population (FAO, 2016). In India wheat provides 50% of the calories consumed, contributing substantially to national food security (DWR, 2014). Global wheat production was 729 Mt in 2014 and in India was 96 Mt (FAO, 2016). India has the second highest global wheat production after China (FAO, 2016). The world population is predicted to reach 9 billion by 2050 and global demand for wheat is predicted to increase at an annual rate of ca. 1.6% yr^−1^ (Godfray et al., 2010; Hall and Richards, 2013). However, the rate of global yield increase in wheat is declining and is currently about 1.1% yr^−1^ (Hall and Richards, 2013). Future increases in grain yield will largely depend on raising above-ground biomass whilst maintaining harvest index (grain DM / above-ground DM; HI). This implies an additional requirement for nitrogen (N) to support increased photosynthesis and biomass production (Reynolds et al., 2012; Aisawi et al., 2015).

Increased N fertilizer inputs will have environmental impacts as well as economic implications. Nitrogen fertilizer has environmental impacts through N leaching and runoff causing eutrophication of freshwater and marine ecosystems and N_2_O (a greenhouse gas) emission associated with denitrification by soil bacteria (Foulkes et al., 2009; Hawkesford, 2014). India is the second largest consumer of nitrogen fertilizers in the world after China (FAO, 2016). Developing new cultivars that have higher grain yield, but which take up and utilize N more efficiently to produce grain will be of economic benefit and help to reduce environmental impacts associated with N fertilizers (Foulkes et al., 2009; Hawkesford, 2014).

N-use efficiency (NUE) can be defined as the grain dry matter (DM) yield (kg DM ha^−1^) divided by the supply of available N from the soil and fertilizer (kg N ha^−1^; Moll et al. (1982) and can be divided into two components: (i) N-uptake efficiency (NUpE; above-ground N uptake per unit N available) and (ii) N-utilization efficiency (NUtE; grain dry matter yield per unit above-ground N uptake). In field studies on historic sets of cultivars under low N supply genetic gains in NUE were mainly related to improvement in NUpE in Mexico (Ortiz-Monasterio et al., 1997) and Finland (Muurinen et al., 2006), but to NUtE in France (Brancourt-Hulmel et al., 2003) and the UK (Foulkes et al., 1998). Under high N supply, NUpE and NUtE explained genetic gains in NUE approximately equally in Mexico (Ortiz-Monasterio et al., 1997), the UK (Foulkes et al., 1998) and Finland (Muurinen et al., 2006), but in Australian NUE gains were more strongly associated with NUpE than NUtE (Sadras and Lawson, 2013). From these studies it is apparent that at low N supply the genetic variation in NUE was more usually associated with NUpE, whereas at high N supply it was more usually associated with NUtE. Furthermore, the effects of N supply and genotype on grain yield often interact. There are to date few studies reporting on genetic variation in NUE and NUE components in modern Indian cultivars under low and high N conditions and its physiological basis.

Mostly selection in wheat breeding is carried out under non-limiting N supply, and plant breeders select cultivars that perform well under optimum N supply conditions. From the above studies, since genotype and N supply do interact, cultivars selected for high yield under high N conditions may not be the highest yielding under lower N conditions. Plant breeding for NUE will require improved understanding of the physiological traits determining higher NUE and responses to N limitation. Promising traits to increase NUE in wheat for which there is reported genetic variation include deeper roots for increased N uptake (Foulkes et al., 2011; Cormier et al., 2016), increased leaf photosynthesis rate per unit N (Gaju et al., 2016), and stay-green traits associated with optimized post-anthesis N remobilization (Gaju et al., 2011; Derkx et al., 2012; Hawkesford, 2014) and/or increased post-anthesis N-uptake (Bogard et al., 2011; Gaju et al., 2014).

A correlation between onset of flag-leaf senescence and grain yield was reported under low N conditions amongst 16 winter wheat cultivars grown at sites in the UK and France, but there was no association under HN conditions (Gaju et al., 2014). Onset of flag-leaf senescence was negatively correlated with the post-anthesis N remobilization efficiency. In a Toisnodor x CF0107 winter wheat doubled-haploid population, pleiotropic QTLs affecting leaf senescence and grain yield and/or grain protein concentration were identified on chromosomes 2D, 2A, and 7D (Bogard et al., 2011). These were associated with QTLs for anthesis date, showing that the phenotypic correlations with leaf senescence were mainly explained by flowering time in this population. Stay-green and fast-senescing mutated (Ethyl methane sulfonate, EMS) wheat lines (*Triticum aestivum* L. cv. Paragon) with similar anthesis date were screened under glasshouse conditions by Derkx et al. (2012) under low N and high N conditions. Delayed senescence was only apparent at higher N supply; in the stay-green line 3, a greater N uptake was observed in the main tiller and the proportion of N in the flag leaf was greater at anthesis compared to the other lines. These results suggest that a better understanding of the mechanisms determining N uptake at anthesis and post-anthesis N remobilization associated with senescence would offer scope to increase grain yield under N limiting conditions and/or combine high yield with high grain protein content in wheat cultivars under optimal N conditions.

The present study aimed to quantify genetic variation in 30 Indian wheat cultivars for grain yield, NUE and NUE components and to investigate its physiological basis with a focus on N accumulation and N partitioning at anthesis, post-anthesis N-remobilization efficiency and senescence-related traits under HN and LN conditions in two field experiments at the Agharkar Research Institute farm in Maharashtra, India.

## 2 Materials and methods

### 2.1 Experimental design and plot management

Two field experiments were conducted at the Agharkar Research Institute farm, near Pune, India (16° 30′ N, 74° 40′ E, altitude: 557.5 m) in 2013–14 and 2015–16. The previous crop was soybean in 2013–14 and sorghum in 2015–16. The soil type was a black cotton soil or Vertisol. The site has a temperate climate with mean annual rainfall of 457 mm and mean annual minimum temperature of 9 °C and maximum temperature of 36 °C. In each year, soil samples were taken before planting at depths of 0–60 cm randomly using a soil corer and submitted for analysis of ammonium and nitrate N and total available N as well as available P and K. Soil mineral analysis showed N, P, K was 197, 25, 578 and 142, 156, 230 kg ha^−1^ in 2013–14 and 2015–16, respectively. The experiments used a split-plot design with two replications in 2013–14 and three replications in 2015–16; the main plot treatment was two levels of N fertilizer application and the sub-plot treatment was 30 elite Indian wheat cultivars. Buffer plots were planted between the N treatment main plots. The sub-plot size was 2.5 × 1.0 m in 2013–14 and 4.5 × 1.5 m in 2015–16. There were four rows 25 cm apart in 2013–14 and 6 rows 25 cm apart in 2015–16.

Two levels of N fertilizer treatment, optimum or high N (HN) and low N (LN), were applied. The high N fertilizer N amount was estimated according to previous knowledge of the site taking into account soil N availability and yield potential using the guidelines of the Indian Institute of Wheat and Barley Research and was 120 kg N ha^−1^ in both years split into two applications. Sixty kg N ha^−1^ was applied at sowing and 60 kg N ha^−1^ at onset of stem extension (GS30, Zadoks et al. (1974)). In the low N treatment, 0 kg N ha^−1^ was applied in 2013–14 and 40 kg N ha^−1^ in 2015–16 at sowing. All N fertilizer was applied as granules of urea (34.5% N) and each split was applied on the same calendar date for the 30 cultivars. All other crop inputs, including pesticide, herbicide and fungicide inputs, and potassium, phosphate and sulphur fertilizers, were applied at levels to prevent non-N nutrients or pests, weeds and diseases from limiting yield. The sowing date was 26 November in 2013 and 2 November in 2015. The seed rate was 240 seeds m^−2^ in both experiments.

Thirty elite Indian spring wheat cultivars were selected. The cultivars were selected to be representative of the most widely grown cultivars in the main wheat breeding zones in India in recent years. All the cultivars were semi-dwarfs except two tall cultivars, Kharchia-65 and BH-1146. There were two durum wheat (*Triticum turgidum* subsp. *durum*) cultivars (PDW- 314 and HI- 8498); the remaining 28 cultivars were bread wheat (*Triticum aestivum* L.) cultivars. Information about the pedigree, year of release and breeding zone origin of the cultivars is given in Table 1.

**Table 1:** The pedigree, date of release and breeding zone of the 30 wheat cultivars in the field experiments in 2013–14 and 2015–16.

Code	Cultivar	Pedigree	Date of Release	Breeding zone^a^
1	BH- 1146	Eonta ponta grossa 1//Fretes/Martin	1987	NEPZ
2	CBW- 38	Cndo/r143//Ente/Mexi-2/3/Ae.squ. (taus)/4/Weaver/5/2*Pastor	2008	NEPZ
3	DBW- 14	Raj 3765/PBW 343	2002	NEPZ
4	DBW- 16	Raj 3765/WR 484//HUW 468	2006	NEPZ
5	DBW- 17	Cmh79a.95/3*Cno79//Raj 3777	2007	NWPZ
6	DBW- 39	Attila/Hui	2010	NEPZ
7	DBW- 46	PBW 343/Inq21	2011	NEPZ
8	DBW- 51	Site/Milan	2010	NEPZ
9	DBW- 71	Prinia/UP 2425	2013	NWPZ
10	DPW- 621-50	Kauz//Altar84/aOS/3/Milan/Kauz/4/Huites	2011	NWPZ
11	GW- 322	PBW 173/GW 196	2002	CZ PZ
12	HD- 2733	Attila/3/Tui/Carc//Chen/Chto/4/Atila	2001	NEPZ
13	HD- 2967	Alondra/Cuckoo//Ures-81/HD-2160-M/HD-2278	2011	NWPZ
14	HD-2932	Kauz/Star//HD 2643	2008	CZ
15	HI- 8498^b^	CR ‘S’-GS’S’//A-9-30-1/Raj 911	1999	CZ
16	HW- 2044	HD 226*5/Sunstar*6/C-80-1	1999	SHZ
17	K- 0307	K 8321/UP 2003	2007	NEPZ
18	Kharchia- 65	Kharchia Local/EG 953	1970	All zones
19	KRL- 19	PBW 255/KRL 1–4	2000	All zones
20	KRL- 210	PBW 65/2*Pastor	2010	NWPZ/NEPZ
21	KRL-1-4	Kharchia 65/WL 711	1990	All zones
22	KRL-213	Cndo/r143//Ente/Mexi-2/3Aeg. sq.(taus)/4/Weaver/5/2*Kauz	2010	NWPZ
23	MACS- 6222	HD 2189*2//MACS 2496	2011	PZ
24	MACS-2496	CM 33027-F-15M-500Y-OM	1991	PZ
25	MACS-6478	CS/TH.SC//3*PVN/3/MIRLO/BUC/4/MILAN/5/TILHI	2014	PZ
26	NW-1067	TR 380-16-3-614/chat’S’	2004	UP
27	PDW- 314^b^	Ajaia12/F3(SEL.Ethio.135.85)//Plat. 13/3/Somat3/4/St./Rsc, 37	2010	NWPZ
28	RAJ- 4229	HW 2048/Raj 4000	2012	NEPZ
29	RAJ- 4238	HW 2021/Raj 3765	2012	CZ
30	WH- 1021	Nyot 95/Sonak	2007	NWPZ

a Northern Hills Zone (NHZ), North Western Plain Zone (NWPZ), North Eastern Plain Zone (NEPZ), Central Zone (CZ), Peninsular Zone (PZ), Southern Hills Zone (SHZ).

b Durum wheat cultivar.

In each year, the crop was irrigated using a gravity based flood irrigation system at 15 day intervals or when irrigation was needed to avoid water stress. The first irrigation was applied immediately after the N fertilizer application at the time of sowing. In 2013–14 the crop was irrigated four times (21 Nov, 26 Nov, 18 Jan, 12 Feb) and in 2015–6 six times (2 Nov, 22 Nov, 9 Dec, 28 Dec, 16 Jan, 3 Feb).

### 2.2 Crop measurements

#### 2.2.1 Developmental stages and plant height

Regular monitoring of crop growth stages was done following the decimal code of Zadoks (Zadoks et al., 1974). In both years, date of anthesis (GS65, AD) and physiological maturity (GS89, 50% of peduncle yellow) was recorded in each sub-plot. Plant height was measured between GS71 and GS89 from the ground level to the tip of ear using a ruler for five randomly selected fertile shoots (those with an ear) per sub-plot in 2014 and three randomly selected fertile shoots per sub-plot in 2016.

#### 2.2.2 Dry matter and plant N% analysis

For dry matter and N analysis, in 2014 five fertile shoots at anthesis and 10 fertile shoots at physiological maturity were randomly selected per sub-plot and cut at ground level. In 2016, samples at anthesis were taken by cutting shoots at ground level in four 25 cm row-lengths giving a sampled area of 1 m^2^ per sub-plot. The fresh weight was recorded and a sub-sample of ten shoots was taken and the fresh weight recorded. At physiological maturity, 12 fertile shoots were randomly selected per sub-plot and cut at ground level.

Shoots were separated into three components: (i) ear, (ii) flag-leaf lamina and (iii) stem, leaf sheath and remaining leaf lamina. Dry weight of each component was recorded after drying for 48 h at 70 °C. Dried ears at harvest were threshed by hand and grain dry weight measured and chaff dry weight was calculated by difference. The number of fertile and infertile shoots was counted in a 1 m row length at physiological maturity in 2014 and at anthesis and at physiological maturity in 2016.

After the samples for DM and N partitioning had been taken at physiological maturity, the remainder of each sub-plot was bulk harvested by hand. The ends of the plots (to 0.25 m) were removed as discard and the rest of the plot was harvested as a bulk by cutting at ground level. The grain was threshed using a threshing machine and weighed and a sample of approximately 50 g grain taken for moisture content analysis. Harvest index (grain DM / above-ground DM) was calculated based on the measurements on the 10- or 12-shoot samples at physiological maturity. Grain number (GN) from the 10- or 12-shoot samples was counted manually and the thousand grain weight calculated (TGW). From these measurements, above-ground DM per m^2^, grains per ear and ears per m^2^ were calculated.

In both years, N concentration of plant components was analyzed by the Dumas method (Dumas, 1831). N% determination was carried out for: i) flag-leaf lamina, ii) remaining leaf lamina and stem and leaf sheath and iii) ears at anthesis (GS65); and for i) flag-leaf lamina ii) remaining leaf lamina and stem and leaf sheath and chaff and iii) grain at harvest. The dried samples were firstly milled into fine powder (particle size <200 μm) using a mixer-grinder mill with mechanical modification for small quantity samples. Milled samples were then re-dried at 80 °C for 48 h and weighed to 0.00001 g precision, encapsulated in tin capsules and analyzed for N%. Nitrogen Nutrition Index (NNI) was estimated according to the ratio of the actual above-ground crop N% at anthesis and the critical N% (N%ct), where N%ct was estimated according to the ‘critical dilution curve’ described by Justes et al. (1994).

Post-anthesis N-remobilization efficiency (NRE) was calculated as the proportion of N in the crop component at anthesis which is not present in the crop component at harvest:

(1) NRE = (N_A_ – N_H_)/N_A_

where NRE is the N-remobilization efficiency of the crop component, N_A_ is the amount of N in the crop component at anthesis and N_H_ is the amount of N in the crop component at harvest.

Post-anthesis N uptake (PANU) was calculated as the difference between above-ground at harvest (AGN_H_) and at anthesis (AGN_A_). In 2014, AGN_A_ was calculated by multiplying the above-ground N per fertile shoot at GS65 by the ears m^−2^ measured at harvest, assuming no change in ears m^−2^ between anthesis and harvest. In 2016, AGN_A_ was calculated by scaling up the above-ground N in the 10-shoot sample based on the FW ratio of the 10-shoot sample and the 1 m^2^ sample at anthesis. The N harvest index (NHI) was calculated as the proportion of above-ground N in the grain at harvest.

#### 2.2.3 Flag-leaf senescence parameters

Senescence kinetics of the flag leaf were assessed visually for main shoots on five tagged plants in 2014 and for the whole flag-leaf layer in 2016 by recording the percentage green area senesced using a standard diagnostic key based on a scale of 0–10 (100% senesced), as described by Gaju et al. (2011). Assessment was carried out weekly after anthesis until full flag-leaf senescence. The data were then fitted against thermal time from anthesis (GS65; base temperature of 0 °C) using a modified version of an equation with five parameters consisting of a monomolecular and a logistic function (Génard et al., 1999) as described by Gaju et al. (2011). The onset of post-anthesis senescence (SEN_ONSET_; °Cd) was defined as the onset of the rapid phase of senescence and the end of post-anthesis senescence (SEN_END_; °Cd) as the thermal time when the visual senescence score is 9.5.

#### 2.2.4 Environmental measurements

Meteorological data were collected daily for rainfall, humidity and minimum and maximum temperature from a meteorological station located 100 m from the field site.

### 2.3 Statistical analysis

Analysis of variance (ANOVA) procedures for a split-plot design were used to analyze N and cultivar effects and test their interaction with year using GenStat version 18 (www.genstat.com; VSN International Ltd, Hemel Hempsted, UK), where replicates were regarded as random effects and cultivar as fixed effects. A cross-season ANOVA was applied to analyze N and cultivar effects across years and the interaction with year, assuming N treatments and cultivars were fixed effects and replicates and seasons were random effects. Pearson’s correlation coefficient and linear regressions were calculated to quantify associations between traits using the 2-year cultivar means using GenStat version 18. Regression analysis with standard linear and curvilinear models (quadratic) was applied to 2-year cultivar means for the 28 bread wheat cultivars to calculate rates of change for traits and grain yield with year of release. Regression coefficients are presented for all variables for the linear regressions. Where quadratic functions increased the proportion of the variance in the variable attributable to year of release compared with the linear function, regression coefficients for the quadratic function are also presented. Bi-plot procedures to test associations between traits were carried out using the R software Version 3.0-2 (http://www.R-project.org/).

## 3 Results

### 3.1 Grain yield, yield components, anthesis date and plant height

**Fig. 1:**
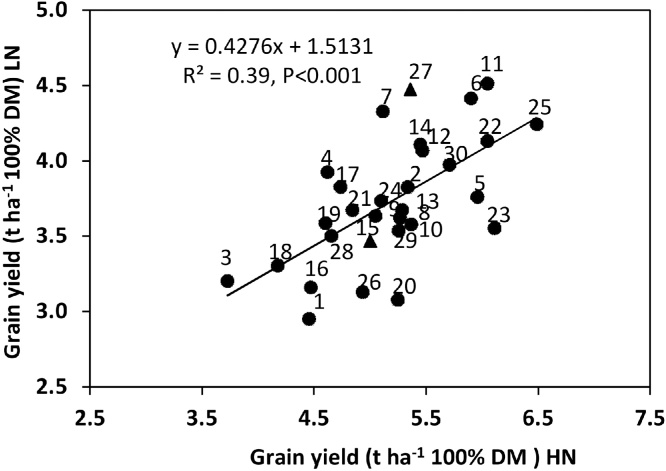
Grain yield of 30 wheat cultivars (codes 1–30, see Table 1 for cultivar names) in high (HN) and low N (LN) treatment. Values represent means of 2013-14 and 2015-16. DF = 174, SED Genotype 0.305***; N × Genotype 0.453* (bread wheat cultivars ●; durum wheat cultivars ▲).

Averaged across cultivars, the reduction in grain yield (GY) in LN compared to HN conditions was 1.49 t ha^−1^ (−29.6%) in 2014 and 1.43 t ha^−1^ (−26.7%) in 2016 (*P* < 0.001; Table 2; Fig. 1), with an average reduction across years of 1.46 t ha^−1^ (−28.1%; *P* < 0.001; Fig. 1). The combined ANOVA for the two experiments showed an interaction between N treatment and cultivar (*P* < 0.05; Fig. 1). On average the proportional loss in GY under LN conditions was less for DBW-46 (0.11), DBW-14 (0.13), K-0307 (0.16) and PDW-314 (0.16) than for DBW-17 (0.36), MACS-6478 (0.37), KRL-210 (0.42) and MACS-6222 (0.42). The year × N treatment × cultivar interaction was not statistically significant. The high tolerance of N limitation for DBW-46, DBW-14 and K-0307 was mainly observed in 2016 and to a lesser extent in 2014, and the low tolerance of N limitation of MACS-6478 was mainly found in 2016. The high tolerance of PDW-314 and the low tolerance of DBW-1, KRL-210 and MACS-6222 of N limitation were generally detected in both years. Overall there was a positive linear relationship amongst cultivars between GY under HN and LN conditions (R^2^ = 0.39, *P* < 0001; Fig. 1). Nevertheless, some cultivars changed rankings. For example, MACS-6222 dropping from 2nd highest under HN to 19th highest under LN conditions; DBW-14 and HW-2044 also changed rankings, ranking relatively higher under LN than under HN conditions.

**Table 2:** Grain yield (100% DM; GY), above-ground N at anthesis (AGN_A_), above-ground N at harvest (AGN_H_), N-utilization efficiency (kg grain DM per kg above-ground N at harvest, NUtE), biomass production efficiency (kg above-ground DM per kg above-ground N at harvest; BPE), post-anthesis N uptake (PANU), grains per m^2^, thousand grain weight (TGW), N harvest index (NHI), grain N percentage, and N nutrition index (NNI). Values are maximum, minimum and means of the 30 wheat cultivars in high N (HN) and low N (LN) treatments in 2013–14 and 2015–16 and the cross-year mean at ARI, Pune, India.

			GY (t ha^−1^)	AGN_A_ (kg N ha^−1^)	AGN_H_ (kg N ha^−1^)	NUtE (kg N kg N^−1^)	BPE (kg N kg N^−1^)	PANU (kg N ha^−1^)	Grains m^−2^	TGW (g)	NHI	Grain N (%)	NNI
2014	HN	Min	3.60	100.9	115	24.4	57.4	−44.10	9002.2	35.64	0.72	2.16	0.84
		Max	5.98	205.5	187	39.6	75.1	35.40	14908	50.42	0.87	2.99	1.27
		Mean	5.03	144.7	149	34.0	68.1	0.57	12064	41.56	0.82	2.41	1.00

	LN	Min	2.38	48.8	53	35.7	79.4	−20.70	4971.8	37.62	0.77	1.63	0.48
		Max	4.84	117.9	114	50.6	102.2	27.30	10206	54.66	0.88	2.27	0.77
		Mean	3.54	78.4	83	43.3	90.4	0.99	7793.6	45.37	0.85	1.93	0.62

2016	HN	Min	3.00	76.6	87	23.9	53.6	−24.30	7730	24.38	0.71	2.07	0.98
		Max	7.25	242.4	233	40.3	89.2	63.20	24356.3	48.64	0.87	2.99	1.49
		Mean	5.35	154.6	175	31.4	67.4	21.90	16404.1	34.02	0.80	2.60	1.19

	LN	Min	2.76	49.7	65	35.7	67.6	−28.90	6866.1	30.45	0.81	1.52	0.60
		Max	5.29	130.7	117	54.1	132.5	23.40	16095.4	47.12	0.86	2.35	0.98
		Mean	3.92	85.3	87	45.9	99.2	3.10	10676.4	37.45	0.84	1.87	0.78

2014-16	HN	Min	3.72	102.8	122	27.2	60.9	−21.40	8782.9	31.37	0.72	2.12	0.92
		Max	6.48	198.1	196	38.2	76.6	46.10	20055.5	49.53	0.85	2.93	1.33
		Mean	5.19	150.6	162	32.7	67.7	13.30	14668.1	37.79	0.81	2.53	1.09

	LN	Min	2.95	62.8	64	37.2	74.3	−17.10	7137.9	36.11	0.82	1.62	0.58
		Max	4.51	111.3	101	51.2	115.6	20.50	12845	50.89	0.86	2.30	0.81
		Mean	3.73	82.8	85	44.6	94.8	2.20	9523.3	41.41	0.84	1.90	0.70

	SED (df)	N (3)	0.16 **	0.76 **	0.51 ***	0.97 ***	9.9 **	0.31 *	383.6 ***	0.16 ***	0.004**	0.04 ***	0.045 **
		G (174)	0.31 ***	1.22 ***	1.12 **	1.75 ***	7.8 ***	0.96 ***	879.4 ***	1.4 ***	0.013***	0.10 ***	0.067 *
		N x G (174)	0.25 *	1.86 ***	0.86 *	1.21 ***	12.8 *	1.4 ns	1281.5 ***	1.9 ***	0.018***	0.15 *	0.103 ns
		YxNxG (174)	0.68 ns	2.69 ns	2.5 ns	7.6 ns	19.4 ns	2.0 ns	1861.7 ns	3.01 ns	0.027ns	0.23 ns	0.151 ns

*Significance at 5% (P = 0.05) level, **1% (P = 0.01) level, ***0.1% (P = 0.001) level and ns, non-significant.

Turning to consider yield components, overall the main effect of N supply was on grains m^−2^ (GN), with a reduction from 14,668 to 9,523 grains m^−2^ (−35.1%; *P* < 0.001; Table 2), rather than individual grain weight which actually showed a moderate increase from 37.8 to 41.4 mg (*P* < 0.001) by the low N treatment (Table 2). The N treatment × cultivar interaction was significant for both yield components (*P* < 0.001). Grains m^−2^ was maintained relatively better under LN conditions for DBW-14 (−19%) and DBW-46 (−20%) than for MACS-6222 (−47.2%) and MACS-6478 (−41.3%). These responses partly explained the GY responses to N treatment. There was also a large range of responses amongst cultivars to decreasing N supply for TGW from −3.4 to +34.1%. Averaging across experiments and cultivars, the effect of N treatment on anthesis date was not statistically significant (*P* = 0.21, data not shown). Overall, AD ranged from 53 days after sowing (DAS) (DBW-16) to 70 DAS (HW-2044 and HD-2733; Supplementary Table 1). Averaging across the two years, there was a positive linear relationship amongst cultivars between AD and GY under HN (R^2^ = 0.18, *P* < 0.05) and LN conditions (R^2^ = 0.30, *P* = 0.002) (Supplementary Fig. 1).

Analysis of variance including breeding zone group as a factor showed that, averaging across years and N treatments, cultivars for the NW Plain Zone and NE Plain Zone groups (65 and 64 DAS, respectively) had later AD than cultivars for the Central Zone (60 DAS) or All Zones (56 DAS) groups, with intermediate AD for the Peninsular Zone group (64 DAS). Although the N × breeding zone effect was not significant for AD, there was an interaction for grain yield. Under LN conditions, grain yields were similar for the PZ, NWPZ, NEPZ and CZ groups in the range 3.69–4.01 t ha^−1^, whereas under HN conditions yields ranged from 4.99 (NEPZ) to 5.93 (PZ) t ha^−1^ for these four breeding zone groups.

### 3.2 N uptake

Overall above-ground N uptake at harvest (AGN_H_) was reduced from 162 kg N ha^−1^ under HN to 85 kg N ha^−1^ under LN conditions (*P* < 0.001; Table 2). Cultivars ranged from 122 (DBW-16) to 196 (MACS-6222) kg N ha^−1^ under HN and 64 (Kharchia-65) to 101 kg N ha^−1^ (HD-2932) under LN conditions (*P* < 0.001;). There was an N treatment × cultivar interaction (*P* < 0.05); DBW-14 maintained N uptake relatively better under LN (-41%) than MACS-6222 (-59%). The three-way interaction with year was not statistically significant. Anthesis date was positively associated with AGN_H_ under HN (R^2^ = 0.30, *P* < 0.01), but there was no association under LN conditions (R^2^ 0.06, ns).

For above-ground N uptake at anthesis (AGN_A_), the main effects of N (*P* < 0.01), cultivar (P < 0.01) and N × cultivar interaction (*P* < 0.001) were significant. The three-way interaction with year was again not significant. Averaging across years, under HN 150.6 kg N ha^−1^ was taken up at anthesis compared to 82.8 kg N ha^−1^ under LN conditions (Table 2). This N accumulation represented 92.9% of AGN_H_ under HN and 97.1% of AGN_H_ under LN conditions. AGN_A_ varied amongst the cultivars from 102.8 (DBW-14) to 198.1 (HD-2967) kg N ha^−1^ under HN and from 62.8 (KRL-210) to 111.3 (DBW-46) kg N ha^−1^ under LN conditions.

MACS-6222 showed a poor capacity for N uptake under LN at anthesis (measured as proportion of N uptake under HN conditions = 0.40). HI-8498 (0.77) and HW-2044 (0.72) showed the best relative N uptakes at anthesis. DBW-14, which showed the best capacity for maintaining N uptake under LN at harvest, also showed high relative N uptake at anthesis (0.62). Later AD was linearly associated with greater AGN_A_ in both the HN (R^2^ 0.40, *P* < 0.001) and LN (R^2^ 0.53, *P* < 0.001) treatments (*P* = 0.003; Table 2).

**Fig. 2:**
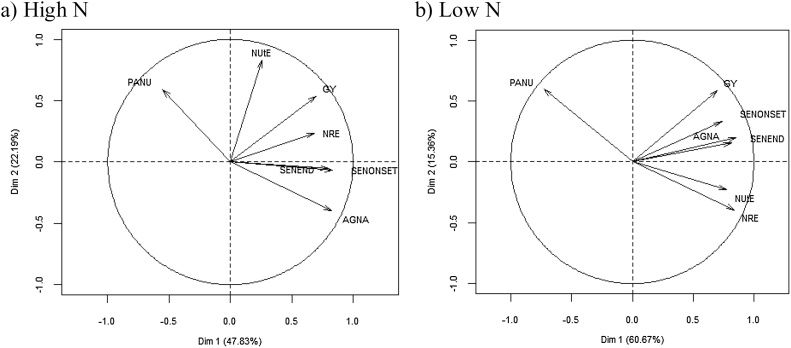
Biplot for grain yield (GY), N-utilization efficiency (NUtE), N remobilization efficiency (NRE), above-ground N at anthesis (AGN_A_), post-anthesis N uptake (PANU), flag-leaf senescence onset (SEN_ONSET_) and flag-leaf senescence end (SEN_END_) in (a) high N and (b) low N treatments for 30 Indian cultivars (mean of 2013–14 and 2015–16).

Post-anthesis N uptake (PANU) was overall relatively low at 13.3 kg N ha^−1^ under HN and 2.2 kg ha^−1^ under LN conditions (*P* < 0.001; Table 2). The cultivars ranged from −21.4 (Kharchia-65) to 46.1 (HI-8498) kg N ha^−1^ under HN and −17.1 (Kharchia-65) to 20.5 (HD-2392) kg N ha^−1^ under LN conditions (*P* < 0.001). PANU was negatively associated amongst the cultivars with AGN_A_ under both HN (R^2^ 0.51, *P* < 0.001) and LN (R^2^ 0.40; *P* < 0.001) conditions (Fig. 2). PANU was also negatively associated with AD under both HN (R^2^ 0.28, *P* = 0.003) and LN (R^2^ 0.34, *P* = 0.001) conditions (Fig. 2).

### 3.3 N-utilization efficiency

Averaging over years, NUtE increased from 32.7 under HN to 44.6 kg DM kg^−1^ N under LN conditions (*P*< 0.01; Table 2). There was a positive linear relationship between NUtE and GY amongst the cultivars under HN (R^2^ 0.26, *P* = 0.004) and LN (R^2^ 0.22, *P* = 0.009) conditions (Fig. 2). There was a N treatment × cultivar interaction (*P*< 0.01); WH-1021 increased NUtE by 16.6% under LN whereas Kharchia-64 increased by 75.1%. There was a positive correlation between AD and NUtE under LN conditions (R^2^ 0.39, *P*< 0.001), but no significant association under HN conditions.

Grain yield can be divided into the components of N uptake (AGN_H_) and NUtE according to:

(2) Grain yield = AGN_H_ × NHI/GPC/(5.7 × 100)

where NHI is the nitrogen harvest index, and 5.7 is the factor to convert grain N concentration to grain protein concentration (GPC). N-utilization efficiency at harvest will be positively associated with NHI and/or negatively associated with GPC according to Eq. (2).

In the present study, NHI overall increased from 0.81 under HN to 0.84 under LN conditions (Table 2). Cultivars differed in the range 0.72 (Kharchia-65) to 0.86 (WH-1021) under HN and 0.81 (KRL-210) to 0.86 (NW-1067) under LN conditions (*P* < 0.001; Table 2), and the response of the cultivars to N limitation differed significantly in the range -1.8 to 12.4%. Differences in anthesis date did not affect NHI under HN, but under LN conditions there was a positive linear association between AD and NHI (*P* < 0.04). Differences amongst cultivars in GPC broadly showed the anticipated inverse association between the higher yielding cultivars and the lower yielding cultivars in the range 13.1% (WH-1021) to 16.4% (BH-1146) under HN and 9.2 (Kharchia-65) to 12.0% (BH-1146) under LN conditions (data not shown). The decrease in GPC under LN differed amongst cultivars in the range 9.5 to 35.5% (*P* <0.001); cultivars with highest GPC under HN conditions tended to decrease by most under LN. There was no association between AD and GPC under HN, but there was negative linear association under LN conditions (R^2^ 0.12, *P* = 0.002).

Averaging across years, there was a positive linear association between NUtE and NHI under HN (R^2^ 0.28; *P* = 0.02) and LN conditions (R^2^ 0.20; *P* = 0.05). In contrast, GPC showed a much stronger negative linear association with NUtE under both HN (R^2^ 0.77; *P* < 0.001) and LN conditions (R^2^ 0.94; *P* < 0.001). Cultivar differences were also observed for the biomass production efficiency (above-ground DM at harvest/AGN_H_; BPE) in the range 60.9 (RAJ-4229) to 76.6 (DBW-39) kg DM kg^−1^ N under HN and 81.4 (DBW-14) to 107.6 (HD-2733) (*P*< 0.01) under LN conditions. The cultivars responded differently to N limitation for BPE with the increase under LN conditions ranging from 18.1 (RAJ-4229) to 36.0 (Kharchia-65) kg DM kg^−1^ N (*P *< 0.05). There was a positive linear association between BPE and NUtE amongst cultivars under both HN (R^2^ 0.33, *P *< 0.001) and LN (R^2^ 0.63, *P *< 0.001) conditions.

**Fig. 3:**
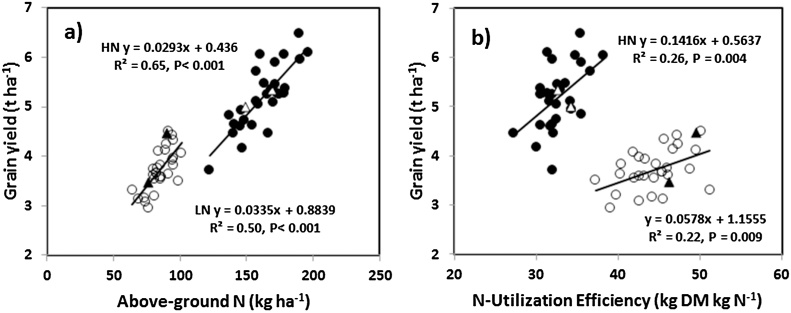
Linear regression of grain yield (100% DM) on (a) N uptake at harvest and (b) N-utilization efficiency (NUtE) under high N (HN) and low N (LN) conditions for 30 Indian wheat cultivars. Values are means of 2013-14 and 2015-16) (bread wheat cultivar HN ●; durum wheat cultivar HN Δ; bread wheat cultivar LN **○**; durum wheat cultivar LN ▲).

Overall AGN_H_ accounted for a greater proportion of the variation in grain yield amongst cultivars than NUtE under both HN (R^2^ values for linear regression of GY on AGN_H_ and GY om NUtE of 0.65 and 0.50, respectively) and LN conditions (R^2^ values of 0.26 and 0.22, respectively) (Fig. 3).

### 3.4 Crop N remobilization efficiency

AGN_A_ was reduced from 150.6 kg N ha^−1^ under HN to 82.8 kg N ha^−1^ under LN conditions (*P* = 0.003; Table 2). Decreases under LN ranged amongst cultivars from 22.9 to 62.3% (*P* < 0.01), but the year × N treatment × genotype interaction was not significant. The post-anthesis N remobilization efficiency increased from 0.79 under HN to 0.84 under LN conditions (*P* < 0.001; Table 3). Cultivars ranged from 0.74–0.84 under HN and 0.78–0.87 under LN conditions (*P* = 0.006). There was no N treatment × cultivar or year × N treatment × cultivar interaction.

**Table 3:** Crop N-remobilization efficiency (NRE), flag-leaf senescence onset (SEN_ONSET_) and end (SEN_END_). Mean, minimum and maximum of 30 wheat cultivars in high N (HN) and low N (LN) treatments (values represent means in 2013–14 and 2015–16).

	NRE		SEN_ONSET_ (°Cd post GS61)		SEN_END_ (°Cd post GS61)	
	HN	LN	HN	LN	HN	LN
Mean	0.79	0.84	849.2	720.4	1057.8	946.9
Min	0.74	0.78	542.5	480.9	874.2	700.4
Max	0.84	0.87	1135.8	940.0	1186.8	1189.7

SED† (df‡)						
N (3)	0.004 ***		15.6 **		27.2 *	
Gen. (180)	0.020 **		62.1 ***		31 ***	
N × Gen. (180)	0.028 ns		87.7 ns		51 ***	
Y × N × Gen. (180)	0.042 ns		126.7 ns		73.3 ***	

† Standard error of the differences of the means. ‡ Degrees of freedom. * Significance at *P* = 0.05, ** P = 0.01, ***0.1% *P* = 0.001 levels; ns, non-significant.

N-remobilization efficiency showed a positive correlation with grain yield under both HN and LN conditions (r = 0.47; *P* < 0.01; r = 0.38; *P* < 0.05, respectively) and AGN_A_ (r = 0.51; *P* < 0.05 and r = 0.35; *P* < 0.10, respectively; Table 3, Fig. 2). N-remobilization efficiency was also positively associated with AGN_A_ (r = 0.42; *P*< 0.05), NUtE (r = 0.40; *P*< 0.05) and SEN_ONSET_ (r = 0.39; P < 0.05) under HN conditions; similar positive associations were also observed under LN conditions (r = 0.59; *P* < 0.001; r = 0.71; *P* < 0.001; and r = 0.45; P < 0.05, respectively). In addition, NRE showed a negative association with PANU under HN (r = −0.41; *P* < 0.05) and LN (r = −0.76; *P* < 0.001) conditions.

### 3.5 Relationships between senescence parameters, yield and NUE components

**Fig. 4:**
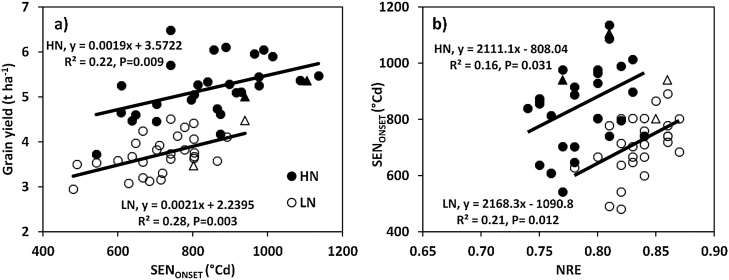
Linear regression of (a) grain yield (100% DM) on flag-leaf onset of senescence (SEN_ONSET_) (b) N-remobilization efficiency (NRE) on SEN_ONSET_ under high N (HN) and low N (LN) conditions for 30 Indian wheat cultivars. (Values are mean of 2013–14 and 2015–16) (bread wheat cultivar HN ●; durum wheat cultivar HN▲; bread wheat cultivar LN **○**; durum wheat cultivar LN △).

Averaging across years, onset of rapid flag-leaf senescence (SEN_ONSET_) was advanced under LN (720.4 °Cd) compared to HN (849.1 °Cd) conditions (*P*< 0.01, Table 3). Under HN conditions, SEN_ONSET_ ranged amongst cultivars from 543 to 1136 °Cd and under LN conditions from 481 to 940 °Cd (*P* < 0.001). SEN_ONSET_ was positively associated with GY amongst cultivars under both HN (R^2^ 0.22, *P <* 0.01) and LN (R^2^ 0.28, *P <* 0.01) conditions (Fig. 2, Fig. 4a). SEN_ONSET_ was negatively associated with NRE under HN (r = −0.46, *P<* 0.05) and LN (r = −0.40, *P* < 0.05) conditions (Fig. 2, Fig. 4b); and positively associated with AGN_A_ under HN (r = 0.58; *P* < 0.001) and LN (r = 0.47; *P* < 0.01) conditions (Fig. 2).

### 3.6 Changes in GY and associated traits with year of release

**Fig. 5:**
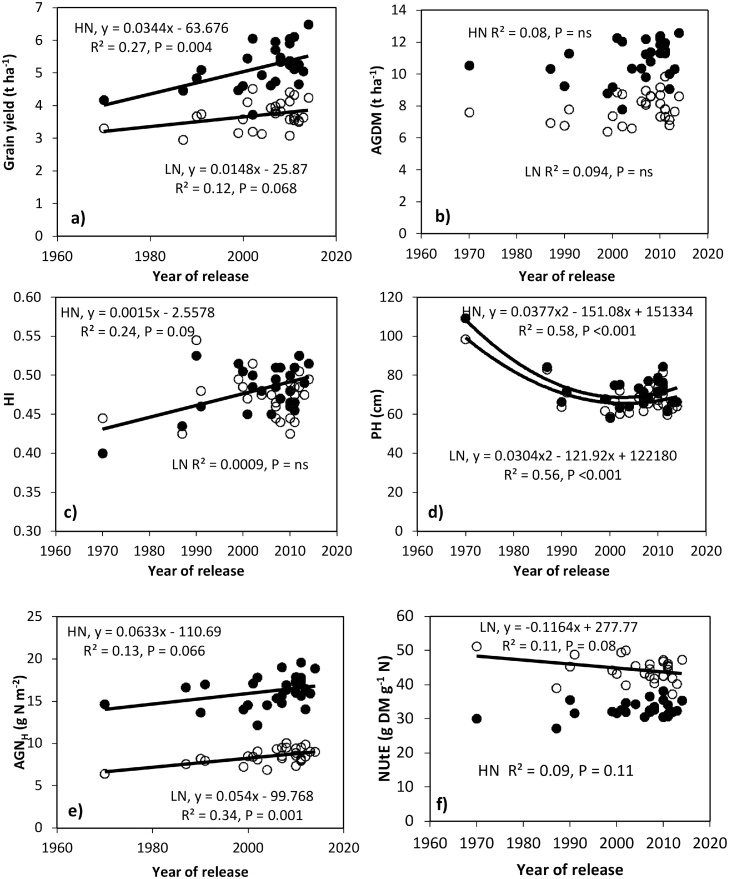
Regression of year of release on (a) grain yield (100% DM), (b) above-ground DM, (c) harvest index, (d) plant height (e) above-ground N at harvest (AGN_H_) and (f) N-utilization efficiency (NUtE) in HN and LN treatment for 28 Indian bread wheat cultivars released 1970–2014 (values are means of 2013-14 and 2015-16). (HN treatment ●; LN treatment **○**).

There was a genetic gain in GY of 34 kg ha^−1^ yr^−1^ under HN and 15 kg ha^−1^ yr^−1^ under LN conditions (0.85 and 0.47% yr^−1^, respectively, relative to GY in 1970; *P* = 0.004 and *P* = 0.068, respectively; Table 4; Fig. 5a). Changes in AGDM_H_ with YoR over the 44-yr period were not statistically significant (Table 4; Fig. 5b). HI increased linearly with YoR over the 44-yr period by 0.0015 yr^−1^ under HN (*P* < 0.01; Table 4; Fig. 5c), but there was no significant increase in HI under LN conditions (R^2^ 0.01, *P* = ns). There was no association between YoR and AD under HN or LN conditions (data not shown). Plant height decreased over the 44-yr period by 30 cm (*P<* 0.01) and 28 cm (*P*< 0.01) under HN and LN conditions, respectively (Table 4; Fig. 5d). The decrease was non-linear with plant height decreasing from 1970 to about 1995 and remaining stable thereafter. Linear genetic gains in AGN_H_ were observed over the 44-yr period of 0.063 N g m^−2^ yr^−1^ under HN (R^2^ 0.13, *P* = 0.066) and 0.054 N g m^−2^ yr^−1^ under LN (R^2^ 0.34, *P* < 0.001) conditions (Table 4; Fig. 5e). NUtE did not change with YoR under HN (R^2^ 0.09, *P* = 0.11), and showed a trend to decrease by 0.116 g DM g^−1^ N yr^−1^ under LN (R^2^ 0.11, *P* = 0.08) conditions (Table 4; Fig. 5f). There was no change in the NNI with YoR under either HN or LN conditions.

**Table 4:** Fitted parameter estimates for changes in crop traits with year of release for 28 Indian bread wheat cultivars released between 1970 and 2014. Linear and quadratic function was fitted to 2-yr cultivar means (2014 and 2016).

Traits		Parameter estimates (y = a + bx)						Parameter estimates quadratic (y = a + bx + cx^2^)						
		y (as in 1970)	b		SE	Pro	y (as in 1970)	c		SE	b		SE	Pro
GY t ha^−1^ (100% DM)	HN	4.01	0.034	±	0.0110 **	0.004								
	LN	3.21	0.015	±	0.0078 †	0.068								
AGDM t ha^−1^	HN	9.60	0.036	±	0.0239 ns	0.143								
	LN	6.93	0.028	±	0.0169 ns	0.113								
HI	HN	0.43	0.002	±	0.0005 **	0.009								
	LN	0.47	0.000	±	0.0005 ns	0.877								
TGW g	HN	33.93	0.100	±	0.0740 ns	0.189								
	LN	36.89	0.121	±	0.0517 *	0.028								
Grains m^−2^	HN	12890.62	56.111	±	55.10 ns	0.318								
	LN	9172.30	10.811	±	27.90 ns	0.701								
PH cm	HN	91.12	−0.549	±	0.1630 **	0.002	108.41	0.03772	±	0.0093 ***	−151.08	±	37.100	<.001
	LN	85.27	−0.491	±	0.1400 **	0.002	99.21	0.03043	±	0.0083 ***	−121.92	±	33.100	<.001
AGN_H_ g N m^−2^	HN	14.04	0.063	±	0.0330 †	0.066								
	LN	6.64	0.054	±	0.0147 ***	0.001								
NUtE g DM g^−1^ N	HN	30.16	0.072	±	0.0436 ns	0.112								
	LN	48.38	−0.116	±	0.0648 †	0.084								
	LN	0.85	0.000	±	0.0004 ns	0.401								
BPE g DM g^−1^ N	HN	69.82	−0.061	±	0.0853 ns	0.478	72.26	0.00534	±	0.0062 ns	−21.37	±	24.700 ns	0.542
	LN	104.13	−0.282	±	0.1520 †	0.075	112.29	0.01781	±	0.0106 †	−71.36	±	42.200 †	0.055
NNI	HN	1.08	0.001	±	0.0018 ns	0.744	1.09	0.00002	±	0.0001 ns	−0.07	±	0.523	0.942
	LN	0.66	0.001	±	0.0012 ns	0.325	0.60	−0.00013	±	0.0001 ns	0.52	±	0.331	0.191

*Significance at the 5% (P = 0.05) level, **1% (P = 0.01) level, ***0.1% (P = 0.001) level, † 10% (P = 0.10) level and ns, non-significant.

## 4 Discussion

### 4.1 The physiological basis of cultivar differences in NUE

Differences in NUE were found amongst the cultivars under both LN and HN conditions. Since the N fertilizer amount applied was the same for all cultivars in a given N treatment, these differences simply reflect differences in GY in a given year/N treatment combination. There was a positive association between AD and GY under both HN and LN conditions. GY increased linearly by 40 kg ha^−1^ day^−1^ with later AD under HN conditions likely related to a longer period for stem elongation increasing radiation capture during the rapid ear growth phase hence grains m^−2^. In the LN treatment, a similar increase in GN with AD was observed, i.e. 40 kg ha^−1^ day^−1^. Since the two slopes were parallel, differences in AD did not contribute to the interaction for GY between N treatment and cultivar. In summary, the differential responses of cultivars to N limitation observed for GY and NUE in the present study were not well associated with differences in AD, e.g. contrasting yield and NUE responses to N supply were observed for MACS-6478 and DBW-14 with the same AD; and also for DBW-17 and DBW-46 with similar AD (Supplementary Table 1).

Present results showed genetic variation in GY was more closely associated with AGN_H_ than with NUtE under both LN and HN conditions. With regard to the association between AGN_H_ and GY, several previous investigations worldwide indicated N uptake accounted for a greater proportion of the genetic variation in GY at LN than at HN supply, e.g. amongst 10 spring wheat cultivars in Mexico (Ortiz-Monasterio et al., 1997), 20 winter wheat cultivars in France (Le Gouis et al., 2000) and >40 spring wheat cultivars in Finland (Muurinen et al., 2006). However, in contrast, our results showed the association between AGN_H_ and GY was slightly weaker under LN compared to HN conditions (R^2^ 0.50 vs 0.65). The reasons for this are not certain. Cultivar differences in rooting traits may partly underlie the association between AGN_H_ and GY. The relatively short pre-anthesis period which varied amongst cultivars overall in the range 53–70 DAS in our experiments may have resulted in a relatively small root system size possibly leading to some restriction in N uptake at depth even under HN conditions in addition to that under LN conditions.

Our results showed a positive association between NUtE and GY amongst the cultivars under HN (R^2^0.26, P < 0.05) and LN (R^2^0.22, P < 0.05) conditions. Moreover, the genetic variation in NUtE was mainly associated with grain N% rather than NHI. An enhanced ability to produce viable grains at a low grain N% may therefore be a trait associated with high NUtE under LN conditions, with the minimum N% in the biomass or grain at harvest influencing how much biomass or grain can be produced per unit of absorbed N. Van Keulen (1977) concluded that the minimum level to which N can be diluted in small grains is approximately 1.6%, and our results showed a minimum grain N% value of 1.61%. However, in winter wheat grown in NW Europe with higher yield potential a minimum value of 1.12% was reported by Gaju et al. (2011) and of 1.26% by Martre et al. (2006). Raising NUtE associated with lower grain N% is feasible in end-use markets for which a high grain starch to protein ratio is desirable, e.g. the feed or biofuel markets. Conversely, improving grain protein composition to maintain grain quality at lower grain N% may be a strategy for improving NUE in cultivars used for bread-making.

Genetic variation in N uptake explained a greater proportion of variation in GY than NUtE under both the HN and LN treatments in our study. This is in contrast to the results of Gaju et al. (2011) in 16 UK and French cultivars and Barraclough et al. (2010) in 39 elite UK winter wheat cultivars which showed that NUtE was the main component determining genetic variation in GY under either low or high N conditions. The cultivar range in NHI in the present study (0.82–0.86) was similar to the study of Gaju et al. (2011) (0.78–0.83) but less than the range of 0.70–0.96 reported by Barraclough et al. (2010). The latter range was likely extended by the inclusion of the older taller varieties Maris Widgeon and Cappelle-Deprez released in 1968 and 1953, respectively, of lower yield potential. However, the cultivar range in grain N% in the present study under LN conditions of 1.6–2.1% was similar to that reported by Gaju et al. (2011) of 1.2–2.0% and Barraclough et al. (2010) of 1.1–1.9%.

The N treatment × cultivar interaction for NUtE was statistically significant with some cultivars increasing NUtE relatively more under low N conditions, e.g., Kharchia-65 and MACS-2496, than other cultivars, e.g. RAJ-4229 and WH-1021. However, overall the grain yield responses to N limitation did not show a correlation with the NUtE responses to N limitation. In contrast, the cultivar responses for AGN_H_ to N limitation were strongly correlated with GY responses to N limitation. For example, DBW-14 and DBW-16 showed large decreases in GY and AGN_H_ under limitation and the converse for MACS-6222 and DBW-17. Therefore, the key to maintaining grain yield well under N limitation in our experiments was maintaining above-ground N uptake. Root traits could partly underlie these differences in AGN_H_ responses as mentioned above. However, due to labour constraints and the difficulty of directly measuring roots (soil coring, root washing and image analysis etc.) root traits were not measured in the present study. Alternatively, N uptake may have been partly determined by the ability to maintain above-ground N demand under low N supply. In this context, stay-green traits will be discussed further below.

### 4.2 Associations between senescence parameters, NRE, NUtE and grain yield

SEN_ONSET_ was positively associated with GY under both HN and LN conditions (i.e, delayed senescence was associated with higher GY). The grain yield of the crops under LN would be expected to be source limited (Gaju et al., 2011), and a positive slope would be anticipated. Under HN most investigations worldwide observed sink limitation or co-limitation of grain yield by source and sink in wheat (Borrás et al., 2004; Acreche and Slafer, 2009) indicating that no association between SEN_ONSET_ and GY might be expected rather than a positive association. The reasons for the positive association between SEN_ONSET_ and GY under HN conditions in our study are not certain. It may be that there was some source limitation of grain growth even under HN conditions. The short season at ARI Pune, Maharashtra with a relatively short duration for pre-anthesis N uptake (on average 63 days under HN conditions) may have resulted in low grain N supply in relation to grain N demand even under HN conditions. Also, post-anthesis N uptake was low under HN conditions in our study at only 13.3 kg N ha^−1^ meaning that grain N had to be supplied almost exclusively from N remobilization from the canopy which may have partly contributed to the apparent source limitation of grain growth.

Present results indicated that the genetic differences in SEN_ONSET_ were partly associated with differences in AD. The association of flag-leaf senescence timing with AD has also been reported previously in wheat (Bogard et al., 2011). However, in the latter investigation the correlation was negative; whereas in our study it was positive (i.e., delayed senescence was associated with later anthesis date). In our study, AD was positively associated with pre-anthesis N accumulation and it seems likely that AGN_A_ was the main physiological driver determining the genetic differences in flag-leaf senescence timings. Previous investigations in sorghum hybrids showed the stay-green trait was linked to changes in the balance between N supply and demand during grain filling resulting in a slower rate of N remobilization from the leaves to the grain compared with senescent genotypes (Borrell and Hammer, 2000; Van Oosterom et al., 2010a,b). Previous investigations in wheat have also found a strong positive correlation between higher pre-anthesis N uptake and the delayed timing of senescence (e.g. Gaju et al. (2014)).

Our results indicated that the NRE was positively associated with GY under low N supply; there was also a positive association between NRE and GY under high N supply. Under high N supply, N saturation probably occurs, and the GY may then be explained by the cultivar’s ability to remobilize N from the straw to the grains, resulting in a higher grain quality and higher GY. Under LN conditions, the reasons for the positive association between NRE and GY we observed are not certain. Gaju et al. (2014) suggested the physiological basis of the stay-green phenotype in wheat was ultimately linked to high grain N supply traits such as shoot N uptake at anthesis which was increased in stay-green cultivars compared to faster senescing cultivars. Our results also overall showed higher SEN_ONSET_ was linked to higher AGN_A_ under LN conditions.

It has been suggested that the stay-green phenotype may be detrimental to the transfer of N to the grain and thus to final grain protein concentration, at least if N uptake does not increase as well (Sinclair et al., 2004). For example, N concentration in straw of a stay-green line remained higher than controls, thus requiring more N uptake to achieve a grain protein content comparable to the wild-type (Chen et al., 2011). In our study, the cultivars with delayed senescence under LN did show a lower grain N concentration than the faster senescing cultivars (R^2^ = 0.23, *p* < 0.001), indicating that the stay-green trait was potentially limiting grain N concentration at maturity.

### 4.3 Implications for breeders

In our panel of 28 bread wheats, grain yield increased linearly with year of release under both HN and LN conditions. These changes were associated with linear increases in N uptake with no change NUtE under HN and actually a trend for a decrease in NUtE under LN. This is general agreement with field studies on historic sets of spring wheat cultivars under low N supply where genetic gains in NUE were mainly related to improvement in NUpE in Mexico (Ortiz-Monasterio et al., 1997) and Finland (Muurinen et al., 2006). In contrast, in winter wheat genetic gains in NUE were mainly related to NUtE in France (Brancourt-Hulmel et al., 2003) and the UK (Foulkes et al., 1998). Sadras and Lawson (2013) found that NNI increased significantly with year of release in Australian spring wheat varieties, hence supporting the conclusion that breeding for yield improved the capacity to uptake nitrogen in equal-sized crops. However, our results did not show a change in NNI with plant breeding.

Currently, in Indian wheat breeding programmes cultivars are selected mostly under high N levels and it may not be cost-effective to select NUE traits under both low N and high N conditions at multi-location trials, although it has been shown that direct selection at LN is more efficient (Brancourt-Hulmel et al., 2005). In the present study, in the low N environments N uptake was associated with genetic progress in GY. In order to improve N uptake, optimizing the rooting system may be an important consideration. Positive associations between increased root density at depth and N uptake in wheat have been demonstrated in field investigations, where variation in rooting traits was due to either different sowing dates and soil types (Barraclough, 1986) or genotypes (Ford et al., 2006), and present findings overall indicated it will be important to consider rooting traits in breeding strategies for improved GY under low to moderate N supply.

Our results showed that identifying genotypes with improved N uptake at anthesis and stay-green traits will be important in future strategies to increase GY in wheat breeding programmes in India under low to moderate N levels. Field screening for physiological traits such as crop N uptake at anthesis and stay-green properties in wheat can be laborious and time-consuming. Several ground-based sensors offer promise for screening large numbers of genotypes for these traits with high precision including those based on measuring spectral reflectance and chlorophyll content (Babar et al., 2006; Araus and Cairns, 2014). Spectral reflectance indices are of particular use in obtaining an objective measure of senescence. In the current experiments, the association amongst genotypes between AGN_A_ measured under LN and HN conditions was statistically significant but weak (R^2^ 0.11, *P* < 0.05), indicating a significant N treatment × genotype interaction. Thus, to be representative of low N performance, assessment of AGN_A_ must be carried out at low N supply. The association for SEN_ONSET_ at LN and HN was stronger (R^2^ 0.72, *P* < 0.001). Further studies are required combining genetics and physiology to identify the location of QTLs for AGN_A_ and senescence-timing traits linked to NUE and to better understand the way in which alleles associated with these QTL may promote substantial differences in NUE in spring wheat.

## References

[bib0005] Acreche M.M., Slafer G.A. (2009). Variation of grain nitrogen content in relation with grain yield in old and modern Spanish wheats grown under a wide range of agronomic conditions in a Mediterranean region. J. Agric. Sci..

[bib0010] Aisawi K., Reynolds M., Singh R., Foulkes M. (2015). The physiological basis of the genetic progress in yield potential of CIMMYT spring wheat cultivars from 1966 to 2009. Crop Sci..

[bib0015] Araus J.L., Cairns J.E. (2014). Field high-throughput phenotyping: the new crop breeding frontier. Trends Plant Sci..

[bib0020] Babar M.A., Reynolds M.P., van Ginkel M., Klatt A.R., Raun W.R., Stone M.L. (2006). Spectral reflectance indices as a potential indirect selection criteria for wheat yield under irrigation this research was partially funded by the Oklahoma Wheat Research Foundation (OWRF), Oklahoma Wheat Commission, and CIMMYT, Mexico. Crop Sci..

[bib0025] Barraclough P. (1986). The growth and activity of winter wheat roots in the field: nutrient inflows of high-yielding crops. J. Agric. Sci..

[bib0030] Barraclough P.B., Howarth J.R., Jones J., Lopez-Bellido R., Parmar S., Shepherd C.E., Hawkesford M.J. (2010). Nitrogen efficiency of wheat: genotypic and environmental variation and prospects for improvement. Eur. J. Agron..

[bib0035] Bogard M., Jourdan M., Allard V., Martre P., Perretant M.R., Ravel C., Heumez E., Orford S., Snape J., Griffiths S. (2011). Anthesis date mainly explained correlations between post-anthesis leaf senescence, grain yield, and grain protein concentration in a winter wheat population segregating for flowering time QTLs. J. Exp. Bot..

[bib0040] Borrás L., Slafer G.A., Otegui M.A.E. (2004). Seed dry weight response to source–sink manipulations in wheat, maize and soybean: a quantitative reappraisal. Field Crops Res..

[bib0045] Borrell A.K., Hammer G.L. (2000). Nitrogen dynamics and the physiological basis of stay-green in sorghum. Crop Sci..

[bib0050] Brancourt-Hulmel M., Doussinault G., Lecomte C., Bérard P., Le Buanec B., Trottet M. (2003). Genetic improvement of agronomic traits of winter wheat cultivars released in France from 1946 to 1992. Crop Sci..

[bib0055] Brancourt-Hulmel M., Heumez E., Pluchard P., Beghin D., Depatureaux C., Giraud A., Le Gouis J. (2005). Indirect versus direct selection of winter wheat for low-input or high-input levels. Crop Sci..

[bib0060] Chen C.C., Han G.Q., He H.Q., Westcott M. (2011). Yield, protein, and remobilization of water soluble carbohydrate and nitrogen of three spring wheat cultivars as influenced by nitrogen input. Agron. J..

[bib0065] Cormier F., Foulkes J., Hirel B., Gouache D., Moënne‐Loccoz Y., Le Gouis J. (2016). Breeding for increased nitrogen‐use efficiency: a review for wheat (*T*. *aestivum* L.). Plant Breed..

[bib0070] Derkx A.P., Orford S., Griffiths S., Foulkes M.J., Hawkesford M.J. (2012). Identification of differentially senescing mutants of wheat and impacts on yield, biomass and nitrogen partitioning. J. Integr. Plant Biol..

[bib0075] Dumas J.B.A. (1831). Procedes de I’analyse organique. Annales de Chimie et de Physique.

[bib0080] FAO (2016). FOASTAT, Food and Agriculture Data. http://www.fao.org/faostat.

[bib0085] Ford K., Gregory P., Gooding M., Pepler S. (2006). Genotype and fungicide effects on late-season root growth of winter wheat. Plant Soil.

[bib0090] Foulkes M., Sylvester-Bradley R., Scott R. (1998). Evidence for differences between winter wheat cultivars in acquisition of soil mineral nitrogen and uptake and utilization of applied fertilizer nitrogen. J. Agric. Sci..

[bib0095] Foulkes M., Hawkesford M., Barraclough P., Holdsworth M., Kerr S., Kightley S., Shewry P. (2009). Identifying traits to improve the nitrogen economy of wheat: recent advances and future prospects. Field Crops Res..

[bib0100] Foulkes M.J., Slafer G.A., Davies W.J., Berry P.M., Sylvester-Bradley R., Martre P., Calderini D.F., Griffiths S., Reynolds M.P. (2011). Raising yield potential of wheat. III. Optimizing partitioning to grain while maintaining lodging resistance. J. Exp. Bot..

[bib0105] Gaju O., Allard V., Martre P., Snape J., Heumez E., LeGouis J., Moreau D., Bogard M., Griffiths S., Orford S. (2011). Identification of traits to improve the nitrogen-use efficiency of wheat genotypes. Field Crops Res..

[bib0110] Gaju O., Allard V., Martre P., Le Gouis J., Moreau D., Bogard M., Hubbart S., Foulkes M.J. (2014). Nitrogen partitioning and remobilization in relation to leaf senescence, grain yield and grain nitrogen concentration in wheat cultivars. Field Crops Res..

[bib0115] Gaju O., DeSilva J., Carvalho P., Hawkesford M.J., Griffiths S., Greenland A., Foulkes M.J. (2016). Leaf photosynthesis and associations with grain yield, biomass and nitrogen-use efficiency in landraces, synthetic-derived lines and cultivars in wheat. Field Crops Res..

[bib0120] Génard M., Reich M., Lobit P., Besset J. (1999). Correlations between sugar and acid content and peach growth. J. Hortic. Sci. Biotechnol..

[bib0125] Godfray H.C.J., Beddington J.R., Crute I.R., Haddad L., Lawrence D., Muir J.F., Pretty J., Robinson S., Thomas S.M., Toulmin C. (2010). Food security: the challenge of feeding 9 billion people. Science.

[bib0130] Hall A.J., Richards R.A. (2013). Prognosis for genetic improvement of yield potential and water-limited yield of major grain crops. Field Crops Res..

[bib0135] Hawkesford M.J. (2014). Reducing the reliance on nitrogen fertilizer for wheat production. J. Cereal Sci..

[bib0140] Justes E., Mary B., Meynard J.-M., Machet J.-M., Thelier-Huché L. (1994). Determination of a critical nitrogen dilution curve for winter wheat crops. Ann. Bot..

[bib0145] Le Gouis J., Béghin D., Heumez E., Pluchard P. (2000). Genetic differences for nitrogen uptake and nitrogen utilisation efficiencies in winter wheat. Eur. J. Agron..

[bib0150] Martre P., Jamieson P.D., Semenov M.A., Zyskowski R.F., Porter J.R., Triboi E. (2006). Modelling protein content and composition in relation to crop nitrogen dynamics for wheat. Eur. J. Agron..

[bib0155] Moll R., Kamprath E., Jackson W. (1982). Analysis and interpretation of factors which contribute to efficiency of nitrogen utilization. Agron. J..

[bib0160] Muurinen S., Slafer G.A., Peltonen-Sainio P. (2006). Breeding effects on nitrogen use efficiency of spring cereals under northern conditions. Crop Sci..

[bib0165] Ortiz-Monasterio R., Sayre K., Rajaram S., McMahon M. (1997). Genetic progress in wheat yield and nitrogen use efficiency under four nitrogen rates. Crop Sci..

[bib0170] Reynolds M., Foulkes J., Furbank R., Griffiths S., King J., Murchie E., Parry M., Slafer G. (2012). Achieving yield gains in wheat. Plant Cell Environ..

[bib0175] Sadras V., Lawson C. (2013). Nitrogen and water-use efficiency of Australian wheat varieties released between 1958 and 2007. Eur. J. Agron..

[bib0180] Sinclair T.R., Purcell L.C., Sneller C.H. (2004). Crop transformation and the challenge to increase yield potential. Trends Plant Sci..

[bib0185] Van Keulen H. (1977). Nitrogen Requirements of Rice with Special Reference to Java Bogor.

[bib0190] Van Oosterom E., Borrell A., Chapman S., Broad I., Hammer G. (2010). Functional dynamics of the nitrogen balance of sorghum: I. N demand of vegetative plant parts. Field Crops Res..

[bib0195] Van Oosterom E., Chapman S., Borrell A., Broad I., Hammer G. (2010). Functional dynamics of the nitrogen balance of sorghum. II. Grain filling period. Field Crops Res..

[bib0200] Zadoks J.C., Chang T.T., Konzak C.F. (1974). A decimal code for the growth stages of cereals. Weed Res..

