# Supplementary material for: Genetic variation in N-use efficiency and associated traits in Indian wheat cultivars
Source: Field Crops Res. 2018 Aug 1;225:152–62. doi: 10.1016/j.fcr.2018.06.002 (PMC6065306; doi:10.1016/j.fcr.2018.06.002)
Supplement: Supplementary file 1 [file mmc1.docx]

**Supplementary Table 1.** Plant height (PH), anthesis date (AD), grain yield (GY), above-ground dry matter (AGDM), harvest index (HI), above ground N at harvest (AGN_H_), N utilization efficiency (NUtE), N remobilization efficiency (NRE), senescence onset (SEN_ONSET_), senescence end (SEN_END_) and grain N (GN) (values represent means of 2013-14 and 2015-16).

|  | PH (cm) | | AD (DAS) | | GY (t ha^-1^) | | AGDM (t ha^-1^) | | HI | | AGN_H_ (g N m^-2^) | | NUtE (g DM g^-1^ N) | | NRE | | SEN_ONSET_ (°Cd) | | SEN_END_ (°Cd) | | Grain N (%) | |
| --- | --- | --- | --- | --- | --- | --- | --- | --- | --- | --- | --- | --- | --- | --- | --- | --- | --- | --- | --- | --- | --- | --- |
| Cultivars | HN | LN | HN | LN | HN | LN | HN | LN | HN | LN | HN | LN | HN | LN | HN | LN | HN | LN | HN | LN | HN | LN |
| BH- 1146 | 84.2 | 83.0 | 55.1 | 54.0 | 4.46 | 2.95 | 10.3 | 6.9 | 0.44 | 0.43 | 16.6 | 7.6 | 27.2 | 39.0 | 0.78 | 0.82 | 702.8 | 480.9 | 961.2 | 785.1 | 2.93 | 2.10 |
| CBW- 38 | 77.2 | 73.0 | 66.4 | 63.6 | 5.34 | 3.83 | 11.4 | 8.6 | 0.47 | 0.44 | 16.9 | 9.5 | 32.6 | 40.4 | 0.74 | 0.81 | 839.5 | 778.0 | 1091.0 | 999.6 | 2.53 | 2.06 |
| DBW- 14 | 63.2 | 60.1 | 54.5 | 53.8 | 3.72 | 3.20 | 7.8 | 6.7 | 0.49 | 0.49 | 12.2 | 8.1 | 32.0 | 39.8 | 0.77 | 0.80 | 542.5 | 666.8 | 874.2 | 700.4 | 2.45 | 2.07 |
| DBW- 16 | 73.4 | 68.6 | 70.4 | 70.5 | 4.62 | 3.93 | 10.4 | 8.3 | 0.45 | 0.48 | 15.4 | 9.4 | 30.5 | 43.3 | 0.75 | 0.84 | 873.8 | 710.7 | 1161.3 | 1189.7 | 2.52 | 1.93 |
| DBW- 17 | 66.2 | 61.6 | 68.6 | 67.4 | 5.96 | 3.76 | 12.2 | 8.1 | 0.50 | 0.46 | 19.0 | 8.3 | 32.0 | 46.0 | 0.80 | 0.83 | 964.8 | 804.0 | 1186.8 | 1029.6 | 2.60 | 1.82 |
| DBW- 39 | 77.1 | 71.2 | 69.4 | 66.7 | 5.90 | 4.42 | 12.4 | 9.2 | 0.48 | 0.49 | 17.1 | 9.4 | 35.5 | 47.2 | 0.83 | 0.87 | 1013.3 | 802.3 | 1097.8 | 1072.2 | 2.32 | 1.82 |
| DBW- 46 | 84.4 | 81.5 | 69.3 | 67.7 | 5.11 | 4.33 | 11.3 | 9.8 | 0.46 | 0.44 | 15.7 | 9.5 | 34.1 | 45.6 | 0.80 | 0.86 | 929.3 | 778.0 | 1031.5 | 1035.8 | 2.39 | 1.84 |
| DBW- 51 | 78.8 | 73.8 | 66.9 | 68.2 | 5.37 | 3.58 | 11.8 | 8.2 | 0.46 | 0.45 | 17.9 | 8.6 | 30.5 | 42.6 | 0.81 | 0.85 | 1086.7 | 865.5 | 1100.4 | 1120.3 | 2.71 | 1.99 |
| DBW- 71 | 66.0 | 63.0 | 58.8 | 57.2 | 5.05 | 3.64 | 10.3 | 7.7 | 0.49 | 0.48 | 15.9 | 9.1 | 32.4 | 40.2 | 0.80 | 0.82 | 804.0 | 803.0 | 1082.1 | 838.6 | 2.59 | 2.09 |
| DPW- 621-50 | 76.3 | 70.2 | 66.5 | 65.0 | 5.27 | 3.62 | 11.5 | 7.8 | 0.47 | 0.47 | 17.8 | 8.0 | 30.7 | 46.1 | 0.76 | 0.86 | 813.5 | 740.8 | 1087.3 | 1023.3 | 2.72 | 1.87 |
| GW- 322 | 75.2 | 72.1 | 68.4 | 66.2 | 6.05 | 4.51 | 12.0 | 8.7 | 0.50 | 0.52 | 17.8 | 9.1 | 34.7 | 50.1 | 0.75 | 0.84 | 856.0 | 740.8 | 1020.0 | 1009.8 | 2.40 | 1.67 |
| HD- 2733 | 74.9 | 66.8 | 70.0 | 70.9 | 5.45 | 4.11 | 12.3 | 8.9 | 0.45 | 0.47 | 17.1 | 8.4 | 32.6 | 49.5 | 0.77 | 0.86 | 976.5 | 891.5 | 1086.1 | 1076.0 | 2.52 | 1.72 |
| HD- 2967 | 74.2 | 72.1 | 70.0 | 70.0 | 5.28 | 3.67 | 11.5 | 7.8 | 0.47 | 0.48 | 17.3 | 8.2 | 31.4 | 45.3 | 0.83 | 0.84 | 898.0 | 804.0 | 1090.6 | 1046.9 | 2.68 | 1.85 |
| HD-2932 | 68.6 | 65.6 | 60.9 | 58.6 | 5.47 | 4.07 | 10.8 | 8.6 | 0.51 | 0.47 | 16.3 | 10.1 | 33.5 | 41.8 | 0.81 | 0.80 | 1135.8 | 803.0 | 1137.1 | 914.6 | 2.52 | 2.04 |
| HI- 8498 Ɨ | 70.3 | 68.3 | 66.2 | 63.9 | 5.00 | 3.47 | 9.8 | 7.2 | 0.51 | 0.48 | 14.9 | 7.6 | 34.2 | 46.2 | 0.77 | 0.85 | 940.0 | 802.3 | 1099.1 | 1013.0 | 2.51 | 1.86 |
| HW- 2044 | 67.9 | 61.8 | 52.5 | 53.2 | 4.47 | 3.16 | 8.8 | 6.4 | 0.52 | 0.50 | 14.0 | 7.3 | 32.1 | 44.2 | 0.75 | 0.82 | 637.8 | 714.8 | 907.3 | 707.8 | 2.67 | 1.85 |
| K- 0307 | 68.8 | 65.6 | 62.7 | 60.2 | 4.74 | 3.83 | 9.8 | 8.2 | 0.49 | 0.47 | 14.8 | 8.5 | 32.5 | 44.6 | 0.75 | 0.83 | 865.5 | 703.7 | 1076.3 | 976.2 | 2.54 | 1.90 |
| Kharchia- 65 | 109.2 | 98.5 | 67.3 | 64.4 | 4.17 | 3.31 | 10.5 | 7.6 | 0.40 | 0.45 | 14.7 | 6.4 | 30.0 | 51.2 | 0.75 | 0.86 | 873.8 | 719.0 | 1022.9 | 920.3 | 2.51 | 1.62 |
| KRL- 19 | 58.2 | 58.7 | 54.8 | 55.3 | 4.60 | 3.59 | 9.2 | 7.4 | 0.51 | 0.49 | 14.5 | 8.5 | 31.7 | 43.2 | 0.78 | 0.84 | 647.5 | 599.8 | 984.0 | 873.8 | 2.59 | 1.97 |
| KRL- 210 | 70.7 | 64.6 | 60.7 | 60.1 | 5.25 | 3.08 | 11.3 | 7.3 | 0.47 | 0.43 | 17.4 | 7.4 | 30.5 | 42.6 | 0.80 | 0.78 | 976.7 | 628.5 | 1177.8 | 877.8 | 2.66 | 1.89 |
| KRL-1-4 | 66.3 | 63.8 | 53.3 | 53.9 | 4.84 | 3.67 | 9.2 | 6.8 | 0.53 | 0.55 | 13.7 | 8.2 | 35.5 | 45.3 | 0.77 | 0.82 | 703.7 | 637.8 | 963.5 | 866.8 | 2.31 | 1.87 |
| KRL-213 | 71.8 | 67.3 | 67.8 | 66.9 | 6.05 | 4.13 | 12.2 | 8.7 | 0.50 | 0.48 | 16.0 | 8.9 | 38.2 | 46.7 | 0.82 | 0.84 | 989.1 | 759.0 | 1104.1 | 1078.3 | 2.12 | 1.79 |
| MACS- 6222 | 72.4 | 65.5 | 62.7 | 61.1 | 6.11 | 3.55 | 11.9 | 7.3 | 0.51 | 0.49 | 19.6 | 8.1 | 31.3 | 44.4 | 0.78 | 0.83 | 888.0 | 666.5 | 1092.2 | 904.7 | 2.59 | 1.90 |
| MACS-2496 | 72.1 | 71.1 | 70.7 | 67.9 | 5.10 | 3.74 | 11.3 | 7.8 | 0.46 | 0.48 | 17.0 | 8.0 | 31.6 | 48.8 | 0.78 | 0.86 | 915.8 | 740.8 | 1082.8 | 1042.4 | 2.64 | 1.81 |
| MACS-6478 | 66.3 | 64.1 | 60.8 | 59.5 | 6.48 | 4.24 | 12.6 | 8.6 | 0.52 | 0.50 | 18.9 | 9.0 | 35.4 | 47.3 | 0.81 | 0.84 | 740.8 | 666.5 | 1043.7 | 900.8 | 2.45 | 1.78 |
| NW-1067 | 64.0 | 60.8 | 63.4 | 61.4 | 4.94 | 3.13 | 10.3 | 6.6 | 0.48 | 0.48 | 14.6 | 6.9 | 34.3 | 45.5 | 0.82 | 0.87 | 795.8 | 684.3 | 1121.8 | 964.3 | 2.40 | 1.88 |
| PDW- 314 Ɨ | 70.9 | 69.6 | 69.8 | 70.1 | 5.36 | 4.47 | 11.3 | 9.3 | 0.48 | 0.48 | 16.8 | 9.0 | 32.9 | 49.5 | 0.81 | 0.86 | 1105.3 | 940.0 | 1134.3 | 1113.1 | 2.65 | 1.73 |
| RAJ- 4229 | 61.5 | 59.7 | 54.6 | 55.1 | 4.65 | 3.50 | 9.1 | 6.8 | 0.53 | 0.51 | 14.1 | 9.9 | 31.9 | 37.2 | 0.76 | 0.81 | 608.3 | 491.0 | 962.8 | 712.3 | 2.54 | 2.30 |
| RAJ- 4238 | 62.3 | 61.7 | 56.1 | 55.8 | 5.25 | 3.54 | 10.0 | 7.1 | 0.53 | 0.49 | 16.5 | 8.5 | 32.0 | 41.9 | 0.76 | 0.82 | 609.2 | 542.5 | 937.8 | 708.1 | 2.48 | 1.95 |
| WH- 1021 | 71.5 | 68.5 | 60.7 | 58.9 | 5.71 | 3.97 | 11.2 | 9.0 | 0.51 | 0.45 | 15.7 | 9.5 | 36.6 | 42.5 | 0.84 | 0.83 | 740.8 | 647.5 | 1016.5 | 906.3 | 2.29 | 1.92 |
|  |  |  |  |  |  |  |  |  |  |  |  |  |  |  |  |  |  |  |  |  |  |  |
| Mean | 72.1 | 68.4 | 63.3 | 62.2 | 5.19 | 3.73 | 10.8 | 7.9 | 0.50 | 0.50 | 16.2 | 8.5 | 32.7 | 44.6 | 0.79 | 0.84 | 849.2 | 720.4 | 1057.8 | 946.9 | 2.53 | 1.90 |
|  |  |  |  |  |  |  |  |  |  |  |  |  |  |  |  |  |  |  |  |  |  |  |
|  | SED† | df‡ | SED | df | SED | df | SED | df | SED | df | SED | df | SED | df | SED | df | SED | df | SED | df | SED | df |
| N | 0.503 ** | 3 | 0.50 ns | 3 | 0.161 ** | 3 | 0.3 ** | 3 | 0.004 ns | 3 | 0.505 *** | 3 | 0.972 *** | 3 | 0.004 *** | 3 | 15.63 ** | 3 | 27.209 * | 3 | 0.043 *** | 3 |
| G | 1.81 *** | 174 | 0.90 *** | 174 | 0.305 *** | 174 | 0.602 *** | 174 | 0.01 *** | 174 | 1.121 ** | 174 | 1.747 *** | 174 | 0.020 ** | 174 | 62.074 *** | 174 | 31.043 *** | 174 | 0.104 *** | 174 |
| N*G | 2.566 ns | 172.3 | 1.30 ns | 84.3 | 0.453 * | 103.2 | 0.89 ns | 113.1 | 0.015 *** | 141.2 | 1.639 * | 129.7 | 2.616 *** | 94.2 | 0.028 ns | 176.94 | 87.714 ns | 174.7 | 51.024 *** | 33.5 | 0.15 * | 141.54 |
| Y*N*G | 3.737 ns | 175.7 | 2.0 * | 53 | 0.681 ns | 90.9 | 1.299 ns | 134.9 | 0.024 ns | 64.2 | 2.46 ns | 104.2 | 3.838 ns | 114.5 | 0.042 ns | 168.98 | 126.71 ns | 180 | 73.336 *** | 62.8 | 0.225 ns | 118.93 |

† Standard error of the differences of the means. ‡ Degrees of freedom. *Significance at the 5% (P = 0.05) level. **1% (P = 0.01) level. ***0.1% (P = 0.001) level and ns, non-significant.

**Supplementary Fig. 1**. Linear regression of grain yield (100% DM) on a) plant height and b) anthesis date (GS65), under high N (HN) and low N (LN) conditions for 30 wheat cultivars. Values represent means of 2013-14 and 2015-16. (bread wheat cultivar HN ●; durum wheat cultivar HN ▲; bread wheat cultivar LN ○; durum wheat cultivar LN (△).
